# Selective emitter using a screen printed etch barrier in crystalline silicon solar cell

**DOI:** 10.1186/1556-276X-7-410

**Published:** 2012-07-23

**Authors:** Kyuwan Song, Bonggi Kim, Hoongjoo Lee, Youn-Jung Lee, Cheolmin Park, Nagarajan Balaji, Minkyu Ju, Jaewoo Choi, Junsin Yi

**Affiliations:** 1 School of Information and Communication Engineering, Sungkyunkwan University, 300 Cheoncheon-dong, Jangan-gu, Suwon, 440-746, South Korea; 2 Department of Energy Science, Sungkyunkwan University, Suwon, 440-746, South Korea; 3 Department of Computer System Engineering, Sangmyung University, Cheonan, 330-720, South Korea

**Keywords:** Crystalline solar cell, Wet etch back, Selective emitter, Acid barrier

## Abstract

The low level doping of a selective emitter by etch back is an easy and low cost process to obtain a better blue response from a solar cell. This work suggests that the contact resistance of the selective emitter can be controlled by wet etching with the commercial acid barrier paste that is commonly applied in screen printing. Wet etching conditions such as acid barrier curing time, etchant concentration, and etching time have been optimized for the process, which is controllable as well as fast. The acid barrier formed by screen printing was etched with HF and HNO_3_ (1:200) solution for 15 s, resulting in high sheet contact resistance of 90 Ω/sq. Doping concentrations of the electrode contact portion were 2 × 10^21^ cm^−3^ in the low sheet resistance (Rs) region and 7 × 10^19^ cm^−3^ in the high Rs region. Solar cells of 12.5 × 12.5 cm^2^ in dimensions with a wet etch back selective emitter *J*_sc_ of 37 mAcm^−2^, open circuit voltage (*V*_oc_) of 638.3 mV and efficiency of 18.13% were fabricated. The result showed an improvement of about 13 mV on *V*_oc_ compared to those of the reference solar cell fabricated with the reactive-ion etching back selective emitter and with *J*_sc_ of 36.90 mAcm^−2^, *V*_oc_ of 625.7 mV, and efficiency of 17.60%.

## Background

The solar cell industry aims to produce high-efficiency solar cells at low cost. The industry has been able to reduce production costs by higher throughput and upscaling of the cell area. One way to reduce solar cell costs is to improve cell performance by applying cheap new methods [1].

Sheet resistance plays an important role in determining the efficiency of a crystalline silicon (C-Si) solar cell because it is related to the surface recombination velocity. The sheet resistance of a common solar cell for commercial applications is about 40 to 50 Ω/sq, which is achieved with homogeneous doping of the emitter region. This doping method can reduce the contact resistance in the metal–semiconductor interface. However, it would increase the surface recombination velocity, and thus, decrease the cell performance [2]. The use of low sheet-resistant emitters in conventional crystalline silicon solar cells usually results in poor short wavelength responses [3]. A lightly doped emitter would provide high sheet resistance and low surface recombination rate, resulting in high internal quantum efficiency in the short wavelength region. However, a lightly doped emitter has a high contact resistance and thus high series resistance [4]. A heavily doped emitter has low contact resistance, but the lifetime of the generated carriers decreases due to the enhanced Auger recombination and Shockley-Read-Hall recombination [5].

To solve the problem of the trade-off between recombination and contact resistance, selective emitter solar cells are introduced. The emitter region where light generated carriers are collected is lightly doped to reduce the recombination velocity, and the emitter region below the contact is heavily doped to reduce the contact resistance [6].

The doping profile of the selectively patterned emitter has historically been obtained by using expensive photolithographic or screen printed alignment techniques and multiple high-temperature diffusion steps [7]. Another way to obtain the doping profile of a selective emitter is to use an etching process such as laser, RIE, or wet etching. The RIE tends to damage the surface, and wet etching does not allow easy control of the sheet resistance [8].

In this paper, selective emitter solar cells are fabricated by the wet etching process. The process is optimized to improve the solar cell efficiency.

## methods

**Figure 1:**
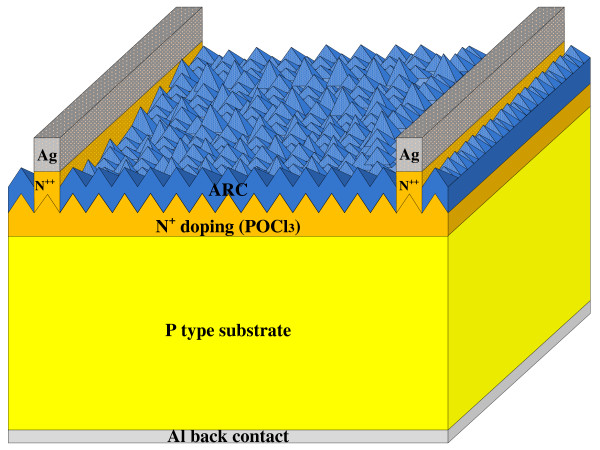
Schematic representation of the wet etched back structure.

The wet-etched back solar cell structure on a large area monocrystalline wafer is shown in Figure 1. The sheet resistance of the portion of the front emitter where light is collected is increased by chemical etching. Lowered surface doping concentration leads to increased sheet resistance. As the dead layer is reduced, surface recombination is also reduced, resulting in improved *J*_sc_ in the region of wavelength below 500 nm. A p-type wafer is doped with POCl_3_. Acid barrier is screen printed and then the chemical etching is carried out to form a selective emitter. After the front and rear passivation with SiNx, Ag is screen printed to form the front electrode and Al is deposited to form the back contact, and then co-firing is carried out.

**Figure 2:**
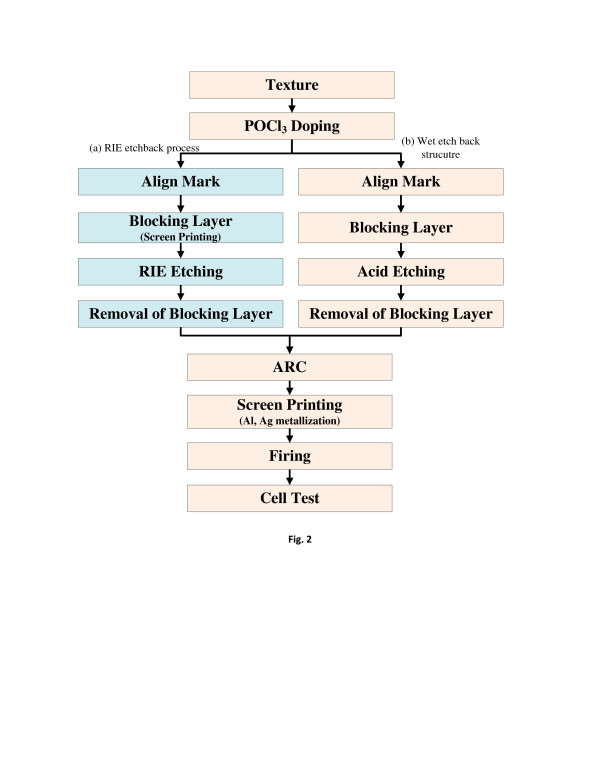
**Processing sequence.(a)** Existing RIE etched back structure using blocking mask, **(b)** wet etched back structure using acid barrier.

The fabrication process of selective emitter structured solar cells is shown in Figure 2. A Czochralski wafer with an orientation of <100>, a thickness of 200 μm, and resistivity of the 1.5 Ω/cm was used. The surface of the wafer was polished with 1% NaOH to reduce the saw damage and then textured by a random pyramid etching process using 2% NaOH and 8.75% IPA. The POCl_3_ doping was carried out in a diffusion furnace at 880°C to obtain a sheet resistance about 30 Ω/sq. The phosphorous silicate was removed by HCl and HF cleaning. For screen printing, an alignment mark was generated by a laser beam and then the acid barrier was screen printed. The acid barrier consisted of 60% of acrylate resin, 20% of TALC, 15% of butyl Cellosolve (The Dow Chemical Company, Midland, MI, USA) and 5% of Solvent Naphtha (Ganga Rasayanie (P) Ltd., Kolkata, West Bengal, India). For comparison, RIE etching, which is one of ways to etch an emitter, was also carried out. For the blocking layer, the mesh pattern for the front electrodes was used in RIE etching. After the RIE etching, the plasma damaged electrodes were removed and the antireflectance coating (ARC) was deposited by plasma-enhanced chemical vapor deposition (PECVD). The front and back electrodes were formed and subjected to co-firing. For the case of wet emitter etching process, the acid barrier was made and then, the lightly doped region was formed by acid etching. The acid barrier paste was removed, and a SiNx layer was deposited by PECVD to use as an antireflection coating.

The rear side metallization was carried out with a standard aluminum paste by screen printing. The front contacts were formed by silver paste screen printing, followed by a firing step at low temperature of 150°C in a belt furnace for the metallization. Illuminated current–voltage (LIV) characteristics were measured under the global solar spectrum of AM1.5G at 25°C.

## Result and discussion

**Figure 3:**
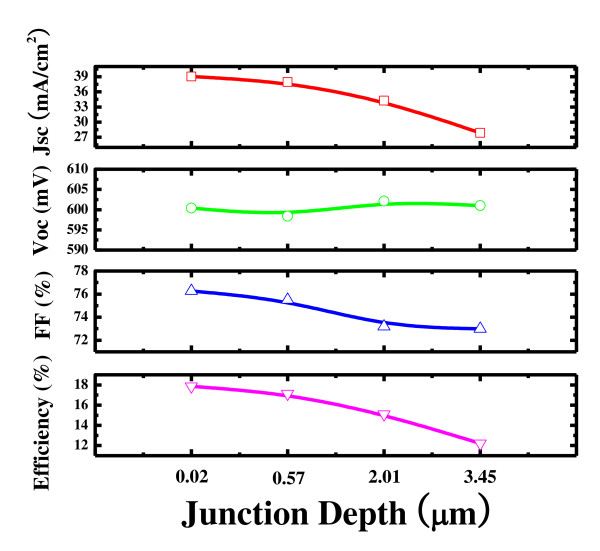
PC1D simulation for selective emitter.

Table 1 shows the cell performance of the monocrystalline silicon solar cells fabricated with RIE process and with wet etch back process. The reference cell, which was optimized in our lab, refers to the general solar cells commercially available. It was processed with 40 to approximately 50 Ω/sq emitter, a standard ARC, and in optimal firing condition. The LIV characteristics of the wet etch back solar cell were as follows: *J*_sc_ of 37 mAcm^−2^, *V*_oc_ of 638.3 mV, efficiency of 18.13%, and fill factor (FF) of 76.77%. Those of the RIE etch solar cell were as follows: *J*_sc_ of 36.9 mAcm^−2^, *V*_oc_ of 625.7 mV, efficiency of 17.6% and FF of 76.4%. The increase in the *V*_oc_ and the improvement of conversion efficiency of the wet etch back selective emitter cell were due to the decreased recombination rate in the emitter and on its surface. The lower *J*_sc_ and *V*_oc_ of the RIE etch back solar cell may be attributed to the degradation caused by damages on the surface of the emitter. The improvements in the cell characteristics of the selective emitter from the advantages offered by the wet chemical etch back process were analyzed by PC1D simulation. Figure 3 shows the electrical parameters of the simulated results. The dopant profiles were analyzed using secondary ion mass spectrometry (not shown in the figure). As the surface concentration is more and shallow junction depth, the number of dopants reduces, increasing the current density as well as the fill factor. Therefore, it can be concluded that while the diffusion profile changes, the higher surface atoms result in the decrease in recombination.

**Table 1:** Light current–voltage results of the reference, RIE etch back, and wet etch back cell

**Name**	***J***_**sc**_**(mA-cm**^**−2**^**)**	***V***_**oc**_**(mV)**	**Efficiency (percent)**	**Fill Factor (percent)**
Reference	35.30	626.7	17.30	78.00
RIE	36.90	625.7	17.60	76.40
Wet etch back	37.00	638.3	18.13	76.77

**Figure 4:**
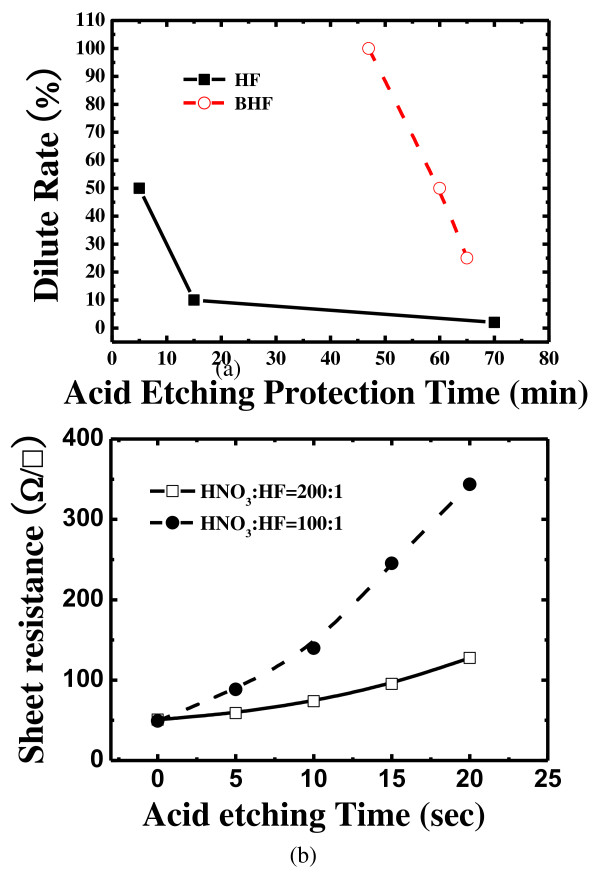
Etching of (top graph) acid etching protection time and (bottom graph) acid etching time.

To form a selective emitter, acid etching was carried out. The region which needed high doping concentration is protected by the acid barrier defined by the blocking layer. Figure 4 (top graph) shows the tolerance of acid barrier paste against etching by various concentrations of HF and BHF. For the case of HF dipping, the acid barrier can withstand etching for 5 min at 49% dilution, 15 min at 10% dilution, and 70 min at 2% dilution. When the guaranteed time is passed, the surface of the wafer becomes affected and the sheet resistance changed. In the case of BHF, the acid barrier can tolerate etching for 47 min at 100%, 60 min at 50%, and 65 min at 25% dilution. It is found that using HF is more effective, for etching, because it shows much lower etching rate than that of BHF. Figure 4 (bottom graph) shows the changes in the sheet resistance as the ratios of HNO_3_:HF and etching times varied. The sheet resistance increases as the emitter etching time is increased. The etching rate is faster for the HNO_3_:HF ratio of 100:1 than for the HNO_3_:HF ratio of 200:1. To use the wet etching process in mass production, it is important to control the sheet resistance as well as the fast etching rate. We used HNO_3_: HF ratio of 200:1 with etching time of 15 s to obtain the sheet resistance of 90 Ω/sq.

**Figure 5:**
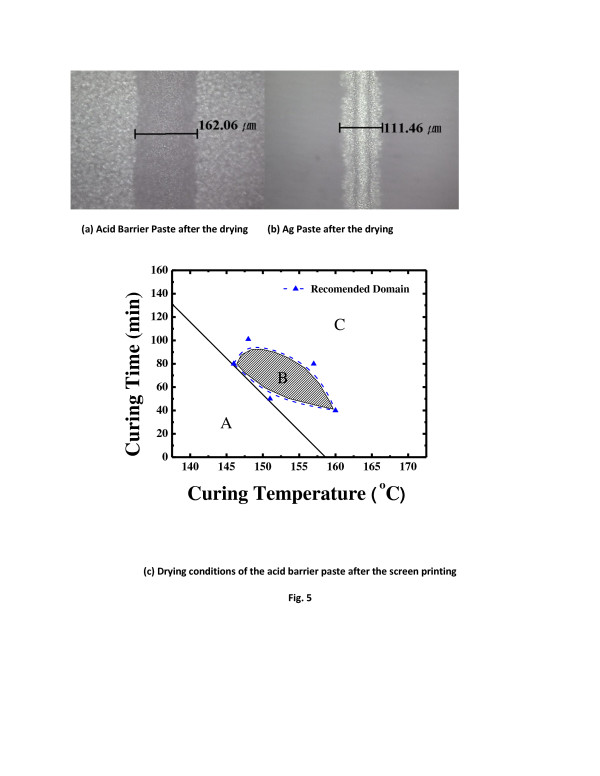
**Optical microscope images(a)** acid barrier paste after drying, **(b)** Ag paste after the drying and curing time **(c)** drying conditions of the acid barrier paste after screen printing.

Figure 5a shows the microscope images of printed acid barrier paste after dying, and Figure 5b shows the screen printed Ag electrodes after emitter etching. The width of the acid barrier paste is 162.06 μm while the width of the front Ag is 111.46 μm. The width of the acid barrier paste was large enough to ensure process margin. When the margin between the highly doped region and the actual electrode was 50 μm, the efficiency loss was 0.05% [9-12]. In our experiment, the width of highly doped region was 162.06 μm and the actual electrode was 111.46 μm, giving a process margin of 50.6 μm.

In the selective emitter solar cell, the sheet resistance around where the electrodes were to be formed was high, which reduces the contact resistance, thus reducing the cell series resistance. To realize a selective emitter, the high sheet resistance region around the electrode should be large enough. Figure 5c shows the drying conditions of the acid barrier paste after screen printing. The temperature and time were varied to find the conditions for properly hardened paste. In region A, the paste is not dried enough and cannot be used as the acid barrier. In region C, the pasted is too hardened and cannot be removed completely after the wet etching. Region B shows the optimal conditions for a selective emitter: drying temperature of 155°C for 70 min.

**Figure 6:**
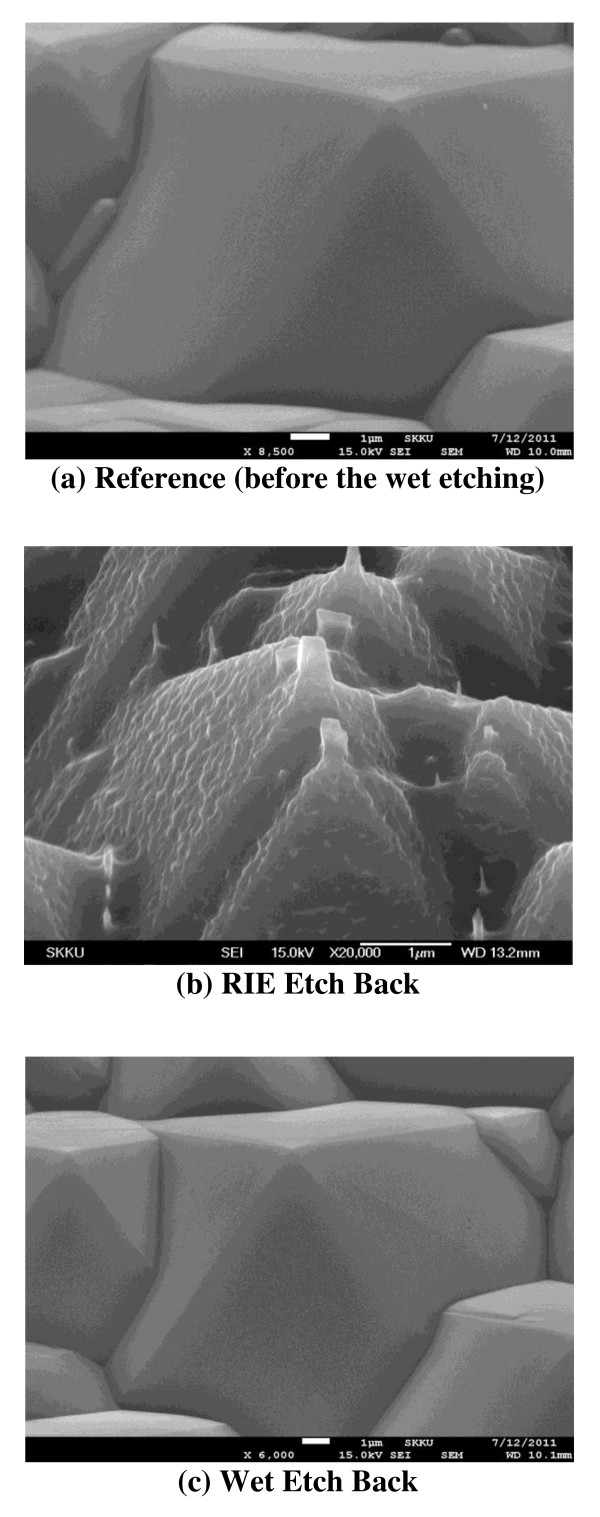
**SEM images(a)** reference (before the wet etching), **(b)**) RIE etch back, and **(c)** wet etch back.

Figure 6 shows the field emission scanning electron microscopy images of textured surface before and after the etching process. The advantages of the wet etching process are as follows: (1) it does not require the use of expensive vacuum equipment and (2) the chemical etching ensures a uniform etching rate and a uniformly etched surface, reducing their defects and increasing lifetime. Figure 6a shows the surface of the conventional reference without the selective emitter. The sheet resistance of the reference cell is 50 Ω/sq. The POCl_3_ doping was carried out at 860°C, and the other process steps, except for the emitter acid etching, were the same. Figure 6b shows the plasma damaged pyramids after the RIE etching. Nonuniform plasma etching resulted in increased surface area and increased number of dangling bonds. Due to the bloated surface, the recombination is expected to increase. The measurement of effective lifetime confirmed the increase in recombination. Figure 6c shows the surface after the wet etching process; the surface is very clean like that of the reference. The effective life of wafers was better after the wet etching than after the RIE etching.

**Figure 7:**
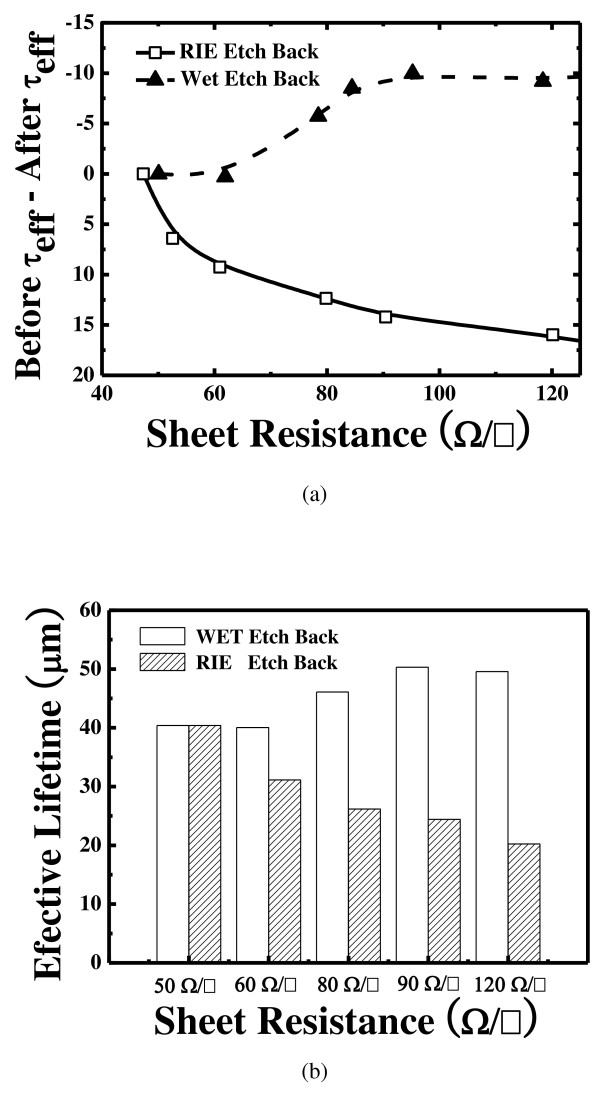
**Comparison of lifetime and sheet resistance between wet and RIE etched emitters(a)** Sheet resistance before τ_eff_ and after τ_eff_ and **(b)** effective lifetime sheet resistance.

**Figure 8:**
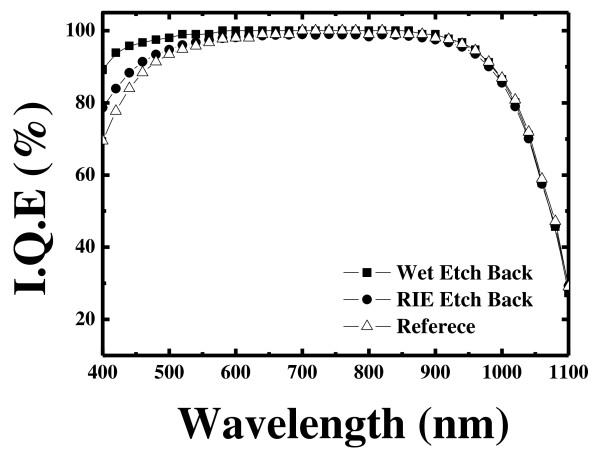
**Spectral responses at different conditions.** The wet etched back with the acid barrier showed enhanced blue wavelength response in the 300 to 500 nm range.

Figure 7 shows the changes in the lifetime for the wet etch back process and the RIE etch back process. The effective lifetime of the wet etch back selective emitter was increased with the RIE etch back process. The internal quantum efficiencies (IQE) of the reference with the RIE etch back and the selective emitter with the wet etch back are shown in Figure 8. The red response (above 700 nm) is the same for both reference and the selective emitter. The excellent blue response of the solar cell with the wet etch back emitter in the shorter wavelength region explains the increased short circuit current density and the more effective diffusion barrier. A better blue response is consistent with the theory that low surface doping concentration lead to less Auger recombination in the emitter region. The RIE process lowered the collection efficiency of photo-generated carriers compared to the conventional process. These defects in the excessively damaged surface can act as recombination centers that decrease the blue wavelength region.

## Conclusions

In this paper, we have presented a new wet etch back selective emitter method that uses the conventional etching paste used in screen printing to control the contact resistance. The HF and HNO_3_ (1:200) solution was used for 15 s to etch the acid barrier, which resulted in high sheet contact resistance of 90 Ω/sq. PC1D simulation was carried out to analyze the cause for the improvements in the cell characteristics of the selective emitter that underwent the wet chemical etch back process. Solar cells of 12.5 × 12.5 cm^2^ with a wet etch back selective emitter were fabricated, achieving an improvement of about 13 mV on the *V*_oc_ compared to those of the reference solar cell fabricated with the RIE etch back selective emitter. The result showed, *J*_sc_ of 37 mAcm^−2^, *V*_oc_ of 638.3 mV, and efficiency of 18.13%, for the cells fabricated with wet etch back; whereas *J*_sc_ of 36.90 mAcm^−2^, *V*_oc_ of 625.7 mV, and efficiency of 17.60% were achieved for the RIE etch back. The wet etch back process gave more uniform and controllable contact resistance with less etching time than the RIE process, and hence, this process can be applied for mass production at a low cost.

## Competing interests

The authors declare that they have no competing interests.

## Authors’ contributions

KS proposed the original idea, carried out the synthesis and analysis of the experiment, and NB wrote the first draft of the manuscript. BK, CP, NB, MJ and JC carried out most of the experiments with KS and shared his idea with the other authors. NB and YJL detailed the original idea and modified the first draft of the manuscript. HL and JY designed and coordinated the whole work and finalized the manuscript. All authors read and approved the final manuscript.
